# Why teach sexuality education in school? Teacher discretion in implementing comprehensive sexuality education in rural Zambia

**DOI:** 10.1186/s12939-019-1023-1

**Published:** 2019-09-27

**Authors:** Joseph Mumba Zulu, Astrid Blystad, Marte E. S. Haaland, Charles Michelo, Haldis Haukanes, Karen Marie Moland

**Affiliations:** 1 School of Public Health, University of Zambia, Lusaka, Zambia; 2 Centre for international Health, Department of Global Public Health and Primary Care, University of Bergen, Bergen, Norway; 3 Centre for Intervention Science in Maternal and Child Health (CISMAC), University of Bergen, Bergen, Norway; 4 Department of Health Promotion and Development, University of Bergen, Bergen, Norway

**Keywords:** Comprehensive sexuality education, Discretion, Schools, Zambia

## Abstract

**Background:**

Reproductive health problems such as HIV, unwanted pregnancy and unsafe abortion among adolescents are closely linked to insufficient knowledge about sexuality and reproduction and lack of access to contraceptives. Supported by international agencies, Zambia has introduced an ambitious nation-wide program for comprehensive sexuality education (CSE) to be implemented into ordinary school activities by teachers. The curriculum is firmly based in a discourse of sexual and reproductive rights, not commonly found in the public debate on sexuality in Zambia. This paper explores how teachers perceive the curriculum and practice discretion when implementing the CSE in mid-level schools in Nyimba district in Zambia.

**Methods:**

Using a case study design, data were collected through in-depth interviews with 18 teachers and analyzed thematically drawing upon theories of discretion and policy implementation.

**Results:**

Individual teachers make decisions on their own regarding what and when to teach CSE. This discretion implies holding back information from the learners, teaching abstinence as the only way of preventing pregnancy or cancelling sexuality education sessions altogether. Teachers’ choices about the CSE program were linked to lack of guidance on teaching of the curriculum, especially with regards to how to integrate sexuality education into existing subjects. Limited prioritization of CSE in the educational sector was observed. The incompatibility of CSE with local norms and understandings about adolescent sexuality combined with teacher-parent role dilemmas emerged as problematic in implementing the policy. Limited ownership of the new curriculum further undermined teachers’ motivation to actively include CSE in daily teaching activities. Use of discretion has resulted in arbitrary teaching thus affecting the acquisition of comprehensive sexual and reproductive health knowledge among learners.

**Conclusion:**

The CSE had limited legitimacy in the community and was met with resistance from teachers tasked with its’ implementation. In order to enhance ownership to the CSE program, local concerns about the contents of the curriculum and the parent-teacher role dilemma must be taken into consideration. Not addressing these challenges may undermine the policy’s intention of increasing knowledge about sexuality and reproduction and empowering adolescents to access contraceptive services and avoid unwanted pregnancies.

## Background

In 2014, Zambia rolled out a new and ambitious framework for Comprehensive Sexuality Education (CSE) targeting children and adolescents enrolled in grades 5–12 in schools across the country [1–4]. In Zambia, sexual and reproductive health (SRH) knowledge is inadequate and unevenly distributed, leading to considerable SRH-related problems among Zambian adolescents [5–7]. Aimed to address such unequal access to knowledge about SRH, the development of a CSE programme was heavily supported by UNESCO [3, 4].

In Zambia, as many as 25% of married girls aged 15–19 have an unmet need for family planning and about 30% of girls aged 15–19 have begun child bearing [5, 7, 8]. Moreover, Zambia has high rates of early marriage with as many as 31% of those aged 20–24 reporting to have married before the age of 18 [5, 8, 9]. While abortions in Zambia are allowed on the broad grounds spelled out in the Termination of Pregnancy Act of 1972, the same law also severely restricts access to safe and legal abortion services by demanding written consent of three medical doctors including a specialist for a legal abortion to take place [10, 11]. This is problematic in a country with critical shortage of health workers. Data on abortion in Zambia is scarce, but recent policy documents from the Ministry of Health estimate that 30–50% of all acute gynecological admissions are caused by abortions and that as many as 6 per 1000 women in reproductive age die from abortion-related causes annually [12, 13]. The problem affects teenage girls in particular; approximately 80% of women taken to health facilities for abortion-related complications are adolescents [9, 14].

Studies in Botswana, Nigeria and South Africa have shown that sexuality education may contribute to overcoming the adolescents SRH challenges that Zambia and other countries face [15–17]. At the core of the Zambian sexuality education policy is the idea that there is a substantial need to support adolescents in delaying their sexual debut, to reduce the number of sexual partners and to increase safer sexual practices [15–17]. Backed by evidence on its positive effects on adolescents’ level of knowledge, skills, attitudes and values related to sex and sexuality, CSE has been promoted in a series of global policy guidelines and recommended to be integrated into ordinary school curricula [3, 15–17]. It is anticipated that the positive effects on knowledge, skills, attitudes and values will empower adolescents to realize their health, well-being and dignity; to develop respectful and pleasurable social and sexual relationships; and to understand and ensure the protection of their rights throughout their lives [18]. Many low income countries have committed to international policies to roll out CSE in their schools [3, 15–17]. Together with 21 other countries, Zambia has signed ‘The Eastern and Southern African Ministerial Commitment on CSE and SRH services for adolescents and young people’ which has shaped expansion and implementation of CSE across the region [1]. This agreement was in turn informed by the International Technical Guidance on Sexuality Education published by UNESCO [18], a guideline that grounds sexuality education within a human rights framework springing out of the CEDAW and the ICPD programme of Action on sexual and reproductive health and rights [18]. The coordination of the development of CSE in Zambia was done by UNESCO [2, 3], and the Zambian framework was developed with continuous reference to UNESCO’s guidelines document. In retrospect, it has been documented that the process of developing and disseminating the content and format of the Zambian CSE was done in a way that left key stakeholders including religious leaders, civic leaders, parents groups and youth without sufficient representation [2, 3]. This may have left the CSE policy without much needed public support.

Zambia has had reproductive health education since the 1990s, but its original content was limited. It did not cover central SRH themes such as gender relations, sexual behavior, information on contraceptive methods as well as values, attitudes, and self-realization life skills which have now been included in the new CSE framework [4]. A key feature of the revised framework is that it is not supposed to be offered as a standalone subject, but is to be integrated in carrier subjects such as science and social studies [4].

Concerns that CSE is incompatible with the religious and cultural norms have been reported to affect acceptability [18]. In Zambia, this is commonly expressed as a conflict between CSE and a tradition of grandparents providing sexuality education along with cultural norms condemning discussions about sexuality between the sexes except for in grandparents-grandchild relations. It is also a common concept that providing sexuality information to young adolescents should be avoided since it will trigger sexual promiscuity. [19]. Similar difficulties in teaching sexuality education have been reported in other countries [20, 21]. Conflicting inter-generational discourses on sexuality between teachers and community members as well as taboos associated with discussion of sexuality [22, 23], and gender-related challenges [23–25], have been reported to affect the acceptability of sexuality education in studies from South Africa and Botswana.

Closely linked to cultural norms and moralization over sexuality are religious values. Zambia was declared a Christian Nation in 1991, a declaration that was included in the preamble of the national constitution [26, 27]. This declaration has given Christian morality a particularly prominent place in Zambian politics and society. It emerges in dominant discourses and weighs heavily in public health discussions about access to reproductive health services to homosexuals, or contraception and safe abortion services to adolescents. This contributes to the conditions causing unequal access to SRH knowledge and services among adolescents [28].

While quite a bit of documentation exists on the challenges of approaching sexuality education in schools in Zambia, there is inadequate knowledge about how teachers handle the task of teaching CSE in schools. This study aimed to investigate teachers’ experiences with the implementation of the CSE curriculum in the Zambian context. We are particularly interested in teachers’ interpretations of their roles in teaching about sexuality, love relations and contraception both vis a vis the pupils and their parents in the community.

In examining the teaching process, we draw upon Lipsky’s theory of ‘street-level bureaucracy’ which relates to the role that frontline workers or ‘street-level bureaucrats’ - such as teachers - play in concrete policy implementation [29]. Street-level bureaucrats are civil servants, or others tasked with the on-the-ground implementation of policies. They function as gate-keepers to services or real-life policy makers since any policy is dependent on health workers, teachers, social workers or others to convert the policy from paperwork to practice. Lipsky notes that, in order to gain enhanced understanding of public policy implementation, one needs to understand that the policy implementation process is dependent on the actions or discretion of those who carry out the policy in actual practice. Discretion, which is the central tenet of the theory, refers to the use of individual decisions or autonomy during policy implementation to vary the quantity and quality of services or information offered to citizens. Discretionary power can also take the form of inaction or resistance to delivering services or providing information [30]. This discretion may be influenced by many issues such as difficulties in making complex decisions [31], limited availability of information and resources as well as when policies are deemed not to be fully compatible with the local context [29, 32]. We used this theory as it is one of the most comprehensive and widely-used theories in understanding bottom-up policy implementation process [32, 33].

## Methods

This study is part of a comparative research project named “Competing discourses impacting girls’ and women’s rights: Fertility control and safe abortion in Ethiopia, Zambia and Tanzania” funded by the Norwegian Research Council and the University of Bergen, Norway [34]. We conducted the study in Nyimba district in Eastern Province of Zambia in 2017. The district was purposively selected as it is one of the provinces with the highest rate of early pregnancies and marriage in Zambia. Primary data were collected by the first author of this paper together with a research assistant at the district level. Designed as a case study of teacher’s experiences of implementing CSE in schools, the study focused on the teachers of six schools, conceptualized as cases and combined in-depth-interviews of teachers with observation of the teaching process and classroom situations. The semi-structured interviews loosely followed an interview guide developed by the first author with input from co-authors. After the first phase of data collection, the results were discussed among all authors and the interview guide was further revised.

A total of 18 teachers were interviewed from six schools in Nyimba district, reaching a level of saturation. We purposively selected the study participants to ensure inclusion of informants with diverse views and experiences about sexuality education. An attempt was made to include teachers across different grades and subjects. The average numbers of hours that the teachers teach varies from about 20 h per week in primary school to about 25 h per week in high school. Classes are made up of about 60 learners. Teacher expertise was largely grouped in two; those who taught basic sciences including mathematics and those who taught social science related subjects such as social studies and religious education. In conducting the recruitment process, we informed the head teachers in the six schools that we were interested in interviewing the head teacher and two other teachers per school (one from the social science- and one from the basic science category). Based on this criteria, teachers discussed and agreed on who would be interviewed for the study. The sample was composed of seven female teachers and 11 male teachers. The male bias was caused by the deficiency of female teachers in some of the schools. The age range of the study participants was from 27 years to 48 years. The data did not suggest that gender, seniority or age had an effect on their experience or forms of engagement with the CSE.

The semi-structured interviews varied in duration between 40 and 55 min and covered the teachers’ experiences with teaching CSE and their thoughts and attitudes towards it.

In addition to the interviews, we also reviewed the Zambian CSE curriculum and other relevant policy documents for documenting their content, framing and approaches used. We analyzed the material using thematic analysis [35], drawing upon Lipsky‘s perspectives on the use of discretion during policy implementation [29]. We focused on developing key themes in decisions about what teachers teach and what shapes their decisions regarding teaching of CSE in schools. The analysis process started with transcribing audio interviews and reviewing the full data set. After a thorough review of the interviews, the development of a code sheet and later coding of the interviews took place. Coding was done using NVIVO version 7 (QSR Australia) which is a qualitative software used to organize qualitative data. The codes were merged into categories, and then themes focusing on forms of discretion and drivers or sources of discretionary power were developed. This was an iterative analytical process which involved: moving between writing themes; reading and analyzing the data; and redrafting the analysis [36–38]. The quotes presented in this text are based on the interviews with the teachers, their experiences being the core focus of the paper.

Ethical approval for the study was obtained from the ERES ethics committee in Zambia (Ref. No. 2017-Mar-003) and the Ministry of Education. Informed consent was given by all participants before being interviewed for the study.

## Results

This section presents findings on the process of integrating CSE into the school curriculum for grades 5–12 in Nyimba district. While the first sub-section describes teachers’ experiences and the dilemmas they encountered in teaching CSE, the second sub-section presents teachers’ reflections on why their role in teaching CSE is problematic.

### Teachers negotiating the comprehensive sexuality education curriculum

We found that the CSE curriculum was treated in an arbitrary manner, leaving much room for the teachers to decide how, when and what to teach as well as what to leave out. With very little guidance, these choices ultimately depended on the individual teacher’s judgement on what would be appropriate to teach considering the time available, the age of the learners and the local norms about sexuality and sexuality education. Their decisions and how they reasoned around their choices is described below.

### This framework does not provide guidance

Teachers were set to teach CSE in grades 5–12, and to integrate the subject into science, social studies, civic education, home economics and religious education. The teachers we interviewed struggled with how this could be done in an appropriate and natural manner and without compromising the attention to and the learning outcomes in the core subjects. Although their knowledge of the content of the CSE in general was incomplete, the teachers shared a feeling of being overwhelmed by the comprehensive list of topics they were expected to integrate into existing subjects: *We are expected to teach relationships, values, attitudes and skills, culture, society and human rights, human development and sexual and reproductive health* (IDI, Teacher 14)*.*

These topics are the same across all grades [5–12], but as the teachers explained they were expected to provide different levels of detail for the different grades:

*In the lower grades, for example Grade 5, on pregnancy prevention, the focus should be on identifying benefits of abstaining from sexual activities while as you go up let’s say to Grades 8, 9 and 10, we are also expected to teach how hormonal contraceptives are used* (IDI, Teacher 2).

The CSE framework describes the expected learning outcomes for each grade, but according to our informants and to our review of the framework, it does not provide guidance on how teachers are supposed to integrate CSE into the specific subjects. This was experienced as both demanding and confusing:

*So when I am teaching home economics or religious education, when and how do I introduce sexuality issues in these subjects? This framework does not provide guidance on such issues. This makes teaching very difficult (*IDI, Teacher 5)*.*

In this void between stated learning outcomes and lack of guidance on how to reach them, teachers were left to solve the problem themselves and make decisions on the integration process on an individual basis as clearly illustrated in this quote: *I decide on my own on what to teach, and how to teach it* (IDI, Teacher 1)*.*

The lack of direction in teaching and integrating CSE has thus implied dependence on individual teachers’ priorities and judgements, and has entailed great variations in the content being taught, when it is taught and how it is taught both within and across schools. Teachers stories show how they moved to amend the curriculum to fit what they perceived to be appropriate through holding back information, emphasizing only some aspects of information or dropping classes on CSE. These tactics or strategies to cope with the problem are explored in detail below.

### Holding back information

Teachers reported being selective about which CSE material they taught and what they left out. *I do not teach them everything, I leave out some stuff* (IDI, Teacher 14).

According to our informants the concept that the school should teach pupils different ways of preventing pregnancy was highly controversial and teachers generally felt very uncomfortable about discussing the wide specter of contraceptive methods listed in CSE and the myths associated with them with learners: *We are expected to discuss types of contraception such as oral contraceptive pill and depo-provera through injectable method, implant, but I do not teach them such things* (IDI, Teacher 10)*.*

To develop the discussion in class into how the different types of contraceptives should be used, and where they could be accessed was perceived as even more awkward and unacceptable:

*Why should I teach about the steps to using contraception? It’s like teaching someone how to fish, they end up always wanting to fish; so in the case of sex, what will happen when they do have contraception, they will still have sex* (IDI, Teacher 8)*.*

The topic on developing good relationships and attributes of a good relationship was seen as difficult to integrate since they did not ‘fit well in subjects such as science and home economics: *When I am teaching science, how do I bring in issues relating to differences between love, affection and infatuation? It does not work for me* (IDI, Teacher 10)*.*

Another reason for leaving this topic out was that teachers feared that it could be misinterpreted by learners as support from the school to engage in romantic relationships.

### Promoting abstinence only

While some teachers would withhold a few selected pieces of the CSE curriculum, others would only agree to teach very limited fragments of it according to what they deemed to be appropriate for learners. Contrary to the philosophy of the CSE curriculum of making information available to adolescents in order to prevent pregnancy, some teachers believed that such information would be counterproductive and decided only to teach one method: *In the school setting, when I teach about preventing pregnancy, the main message is only about abstinence* (IDI, Teacher 16)*.*

Hence, most of the time available was dedicated to abstinence and the benefits of abstaining from sexual activities. The very strong moral message on abstinence was put across in several ways:

*We have a song about the importance of abstinence, we sing it before we start each session on CSE. I also tell them repeatedly that abstinence is the only method that can help them avoid teen pregnancies and STIs including HIV* (IDI, Teacher 14).

This group of teachers saw their role as much as one of preventing sexual activity among their pupils as one of preventing pregnancy. They reported to prioritize their time teaching about the importance of avoiding exposure to situations that could tempt pupils into sexual stimulations or encounters: *I tell them to avoid intimacy, being with someone of the opposite sex in a secluded place or watching things that will make them think of the opposite sex* (IDI, Teacher 3)*.*

### Dropping topics

Other teachers dropped topics or defined sessions in the CSE altogether. This took different forms within and between the schools. Interviews with teachers showed that some of them substituted the whole CSE topic, which they were not comfortable teaching, with other topics which they believed were more appropriate for learners*.* A teacher told us how he substituted a topic with another:

*I skipped the whole topic on pregnancy prevention. Instead of teaching about condom use, I moved to another topic. I repeated sessions which I thought were good for learners such as communication, assertiveness and decision-making skills* (IDI, Teacher 9)*.*

In some cases, when teachers had already taught the topics in the CSE they were comfortable with or felt were appropriate, they turned to teaching completely different subjects with little relevance to the CSE curriculum: *When I realized that I had taught all topics that I was comfortable with, instead of teaching topics on condoms or oral contraception use, I decided to only teach social studies* (IDI, Teacher 9)*.*

Teachers that were most reluctant to teach CSE could even took a more radical step to avoid teaching. Some told us that when it was time for them to teach CSE, they sent learners to do outdoor activities which were not related to CSE:

*I opt to send the students out for sport activities, preventive maintenance work and other club activities instead of teaching CSE* (IDI, Teacher 5)*.*

Interviews with teachers showed that teaching CSE was not done on a routine basis and in a standardized manner and that the CSE curriculum was treated haphazardly in the schools. Teachers could not state on average how often they taught CSE and a few teachers reported that they had stopped teaching CSE altogether and as one teacher put it;

*All I can say that it is something that happens by chance* (IDI Teacher 7).

### Lack of local ownership of the CSE agenda

The decision-making process among teachers regarding when, what and how to teach comprehensive sexuality education was informed by a number of factors. The reasons, which we outline in detail in this section, included perceived incompatibility of CSE with the local culture, teacher-parent role dilemma, concerns about the legitimacy of the CSE concept and practical challenges related to lack of training and access to manuals.

### Incompatibility with the local culture

Comprehensive sexuality education was seen as incompatible with the local culture and religious values, as it confronts local ideas about sexual morality. There were concerns that some topics were too sensitive as they were believed to promote pre-marital and casual sex among learners. *We are a Christian country, so the message for us is no sex before marriage* (IDI, Teacher 9)*.*

Another teacher explained how provision of CSE information would promote casual sex:

*Many children avoid sex because of fear of pregnancy. No, if they know that they can prevent pregnancy by using contraception, children may get too excited and confident, and start getting involved in casual sex* (IDI, Teacher 7)*.*

Teachers further reported that parents were against the teaching of some components of CSE in schools as they considered CSE topics to be sacred only to be taught by traditional counsellors at community level:

*The controversy is also about the place where such information is delivered from not being culturally appropriate, it’s taboo to teach sexuality education in a school* (IDI, Teacher 11)*.*

At one school, a teacher narrated an event which had caused uproar from the community. In an effort to implement the CSE curriculum, the teacher had asked learners to do an exercise at home on initiation ceremonies for girls when they reach puberty:

*Having seen the assignment, which I gave to the learners, parents came in numbers to the school in the morning and demanded to see the headmaster. I was called to attend the meeting. The parents then complained to the headmaster that the initiation ceremony is something special which should not be handled at the school* (IDI, Teacher 6)*.*

The notion of sexuality education as sacred and belonging to arenas of learning very different from the school surfaced strongly and placed teachers in some squeeze vis a vis the parents. A complicating factor was gender mixed classes. It was highly uncustomary to discuss sexuality and reproductive health issues specific to female or male learners in the presence of the opposite sex. Adding to the problem was age. Customarily sexuality education was not supposed to be introduced to children in the lower grades. It should be introduced only during the initiation ceremony taking place later after girls attain puberty, and many teachers shared this understanding with the community and had difficulties discussing sexuality issues and using sexuality terminology particularly with the youngest learners.

As the young students were not conversant in English, the classes on CSE had to be provided in the local language which was experienced by the teachers as more challenging since the local terms emerged as more insulting than the English ones. In order to cope with the embarrassment teachers used different strategies. As one of them explained:

*I close my eyes when I mention the sex organs* (IDI, Teacher 1).

The taboo related to mentioning sex organs in the local language in teacher-student discussions was clearly expressed in the practice of giving the teachers insulting nicknames. As a way of avoiding antagonism with the community, teachers reported leaving out or omitting issues that they perceived as inappropriate from the community perspective.

### Teacher-parent role dilemma

The dissenting or opposing views from the community about teaching sexuality education in the school, coupled with cultural and religious values about morality presented a professional challenge for teachers. On the one hand, they were supposed to convey knowledge and stimulate reflections as described in the curriculum. On the other hand, teachers were expected to have a broader role vis a vis their pupils bringing them up according to social and cultural norms and values. Teachers reported that they struggled to strike a balance between teaching sexuality education to their pupils and maintaining the broader parental role of shaping them into responsible adults:

*It is very difficult for me. As a parent I need to promote abstinence, but as a teacher this curriculum wants me to talk about the importance of using condoms. One topic for example requires us to describe the steps that one has to follow when using a male or female condom. Now, how do I demonstrate such steps to learners who are almost the same age as my child? No, that’s like teaching children to be ‘sex experts’* (IDI, Teacher 7).

This situation was even more challenging for teachers who had biological children in their class and bolstered the tendency to skip CSE sessions on sensitive topics: *I think about my children, so when I know that the topic is not good for them, I skip the topic* (IDI, Teacher 9)*.*

Because of this role dilemma, other teachers suggested the need to think about other approaches to delivering CSE. One recommendation was to engage other actors to deliver CSE: *Some topics can be taught by teachers and other topics can be taught by people outside the school such as health workers or community health workers* (IDI, Teacher 7).

### Concerns about the legitimacy of the CSE concept

Concerns about the legitimacy of the CSE concept also emerged throughout the interviews. Many teachers reported not being comfortable teaching CSE as they considered it to be something that was externally driven with little relevance to local needs. When we asked one teacher why some teachers have stopped teaching CSE, he seemed to perceive it as a foreign agenda: Y*ou mean this donor funded program, some teachers have sidelined sexuality education, it’s just extra work for us* (IDI, Teacher 13).

In addition to cultural incompatibility, the inadequate involvement of actors at the district level during the development, validation and dissemination process affected the legitimacy of the curriculum. Some teachers argued that instead of offering CSE, the community would have preferred more topics that directly address poverty-related issues:

*They (developers) should have known that this one is a hot issue. It is not as simple as introducing a new curriculum for social studies or science. This one (CSE) touches on what people believe in, people’s culture and how people bring up children. To make it even more complicated, we did not cover it during the training process in college, thus as people that are supposed to implement it, we should have been consulted* (IDI, Teacher 14)*.*

### Limited prioritization of sexuality education

Teachers reported that, compared to other subjects, CSE implementation was weak and was characterized by several severe gaps, including lack of adequate training of the teachers involved: *The headmasters attended a 2 days training in CSE, and then they briefed teachers in schools on CSE for only one to two hours. So how do you expect us to effectively teach?* (IDI, Teacher 2).

Lack of teaching aids or images and reading materials in schools was another gap and was seen as particularly important for explaining complex and sensitive topics: *We also need images to explain for example topics on unsafe abortion, cancer, STIs. For now, we have to borrow images from the health facility* (IDI, Teacher 4).

Teachers also had challenges accessing the manuals as only one manual was given to each school: *The headmaster locks the only copy in his office. So how do we teach?* (IDI Teacher 4).

Furthermore, the topics in CSE were not reflected in the common scheme of work which all schools in the district were supposed to teach: *After the schemes were completed, then we just realized that we accidently left out comprehensive sexuality education* (IDI, Teacher 1).

While some kind of teaching of CSE is going on in the schools in the district, many teachers were grappling with the puzzle of why CSE was introduced. This was primarily related to the weakness that has surrounded the implementation of CSE compared to other subjects: *We have been teaching social studies for a long time and at no point did we see parents come and protest about the topics, so why should we continue teaching something (CSE) that the community has concerns over?* (IDI, Teacher 5).

The decision by the Government to implement CSE without providing adequate support in schools made some teachers question the timing of the implementation process: *My question is about why they (Government) decided to implement CSE when they were not ready. I always wonder what caused this rush?* (IDI, Teacher 14).

The puzzlement among teachers about the rationale for introducing CSE made them question why they have to teach CSE. This lack of appreciation of teaching the new CSE framework by teachers is best illuminated in the following question raised by a teacher when we her asked why she had stopped teaching sexuality education: *If I may ask*, *why do they want us to teach sexuality education?* (IDI, Teacher 7).

## Discussion

The study has examined teachers’ interpretations of their role in teaching sexuality, love relations and contraception during the early phase of implementation of CSE in a rural district in Zambia. We have noted that what, when and how to teach is dependent on the individual teachers’ decisions. In line with Lipsky’s [29] call for the need to move beyond the top-down approach to policy analysis, − and consider other contextual realities that shape policy implementation - our study strongly demonstrates how the settings within the schools in which the CSE framework was implemented influenced how teachers made decisions about the curriculum and subsequently the pattern and nature of the implementation of CSE. This study’s findings revealed that the lack of clarity in the CSE framework, on how to integrate CSE teaching into existing subjects, coupled with contextual challenges, left teachers involved in CSE with a great room for discretion. In this context, extensive use of discretion resulted in arbitrary and unequal management of the CSE curriculum in the district. Lipsky notes that unclear or vague policy guidance as well as features of work settings or context in which street-level bureaucrats or workers act can make the bureaucrats interpret and implement the policy content in different ways [29]. According to the theory of street-level bureaucracy, the differential policy interpretation happens because the lack of clarity in the policy gives the implementers space and power to exercise individual discretion in interpreting the content and direction of the policy [33].

In this study, some of the features of work settings that shaped decision making among teachers were socio-cultural factors. These factors included incompatibility of CSE with the local culture and religious ideals. For example, while the CSE framework required teachers to discuss different ways of preventing pregnancy, religious and cultural values expected teachers only to focus on abstinence. Such incompatibility created teacher-parent role dilemmas in the classroom setting. Teachers tended to see themselves in a parental role with the obligation to shape their pupils into responsible or morally upright adults. Abstinence was a key message in this regard. The setting had inadequate support for CSE such as inadequate training, materials and tools for teaching as well as insufficient leadership and guidance in the implementation process. These gaps made some teachers question the extent to which CSE is prioritized within the education system and why they have to teach sexuality education. We note that such doubts among teachers potentially provided more space for discretion, and subsequently enhanced the power teachers had to skip some aspects of CSE or not teach it at all. These study findings, like other studies that have discussed the concept of discretion, agrees that the application of discretion or autonomy during policy implementation is potentially also motivated by the availability, or otherwise, of resources [29, 32, 39, 40].

As a way of dealing with these dilemmas and the gaps in support, teachers modified their teaching of CSE, a practice that is articulated in street-level bureaucracy theory. According to this theory, when they are faced with challenging situations, bureaucrats use their discretion to modify how they understand and execute their tasks or responsibilities [29, 33]. Lipsky [41] notes that this invention or modification of modes of decision is done in order to serve the “workers’ agency or purposes”(p.xiv). This modification of policy content, which is also known as coping, can happen in three forms. The forms include: bureaucrats’ adjusting or moving towards clients through bending policy options in order to meet the needs of clients; moving away from clients or rationing services; and moving against clients through rigid application of rules [42]. In our case study, the modification process adopted by teachers in relation to their clients (pupils and parents) was moving towards clients by bending the CSE policy.

This study further showed that the use of discretion to modify what to teach was justified by teachers as the best way to protect the children from sexual harm. Teachers feared that some information would motivate learners to engage in sex as they would no longer have to worry about pregnancy. They argued that the situation had the potential of turning the learners into ‘sex experts,’ putting them at risk of pregnancies in cases where there is no contraception or contracting an STI if condoms were not available. This process of exercising discretion among the teachers in the district was motivated by p*aternalism, as they viewed or defined learners as*, “children in need of protection, rather than as young people who have the right to relevant information about their own bodies and their sexuality*”* [43], p. 36). As described above, teachers justified adopting paternalistic values as they perceived themselves as ‘parents’ of all children in the class. It is important to note that paternalism was further articulated through resistance towards teaching CSE. Teachers resisted teaching CSE as they viewed it as something that was externally driven with little relevance to local needs as well as incompatible with the cultural norms and values.

The actual policy that is realized vis a vis clients depends more on those who carry out the policy than the policy makers [39]. In the context of this study, implementing CSE is a *‘negotiative process’* between the teachers and the contextual realities such as the broader educational system, socio-cultural and community dynamics, as well as the experiences and values of individuals. We note that the agency and power among the workers, in this case the teachers, coupled with interactions between teachers and the school environment influenced the implementation of the policy [44, 45] which resulted in unequal access to CSE among learners. In our case, these powers included holding back some CSE information, teaching only abstinence and dropping classes. These scenarios, therefore, make the outcome of the policy implementation process a result of the complex interplay or interaction between the frontline workers and the contextual realities. Lipsky [29] refers to this phenomenon – interaction and negotiating process- as “a gap between policy as written, and policy as performed” (p. xvii). We further note like Lipsky’s(29)words, that as teachers interact and negotiate during the implementation process of CSE, “the routines they establish, and the devices they invent to cope with uncertainties and work pressure, effectively become the public policies they carry out” (p. xii). We therefore agree, based on the findings on this study, with Gilson’s [33] view that for “all bottom uppers, policy-making is still in progress at the moment of delivery” (p,9).

Meanwhile Lipsky [41] cautions that negotiations during the policy implementation process and subsequent policy modifications “may widen the gap between policy as written and policy as performed” (p.xvii). Thus although “discretion” may promote teachers’ freedom to tailor and adapt their teaching to the needs of their pupils, it may lead to widening the gap between policy as stated and practiced. This widening gap between policy and practice may distort service ideals [33]. For example, adopting *paternalistic approaches in delivering CSE may affect* the acquisition of skills about reproductive health among young people. Paternalism may affect learning: learners may not be or feel able to ask questions freely on sensitive topics such as contraception use because of the limited interaction and lack of frank discussion between teachers and learners and the moralizing context of sexuality education [46]. This may negate the very essence of establishing CSE in such communities and perpetuate the absence of critical knowledge and life skills to prevent early pregnancy. In a country with high pregnancy and early marriage rates, this lack of knowledge is problematic. Our findings resonate with other studies that have examined the use of discretion in delivering welfare and prison services, ie, that nonconformity to prescribed policies by street-level bureaucracy can lead to disparities in access to services for some populations [31, 47].

Improving policy implementation requires paying attention to the contextual realities that reinforce discretion during this process [30]. As we have discussed above, policy as experienced by clients is a reflection or a product of the interplay between both the formal and informal practices of street level bureaucrats [48]. Enhancing the implementation of CSE may require increased involvement of stakeholders at local level in developing and implementing CSE policies and programs, as well as providing comprehensive training in CSE to teachers. As observed in this study, the limited involvement of local actors made teachers see CSE as a foreign agenda which was not compatible with their local context or their mandate to teach. Other authors on CSE in Nigeria and a recent publication on international cooperation in sex education have also cautioned that limited involvement of local actors has the potential of developing CSE which is insensitive to local collective concerns and networks [49, 50]. We stress the need for giving stakeholders at the lower level (policy implementers) a much bigger role in developing the content and implementation strategy of CSE as they have better knowledge of the context, networks and local support which they can use to negotiate or navigate micro level politics.

### Concluding remarks

We conclude that the implementation of the CSE curriculum in this setting was largely dependent on an individual teacher’s decisions on what, how and when to teach. This was related to lack of guidance, lack of legitimacy of the curriculum, and lack of local ownership of the agenda. The big space left for teacher discretion in sexuality education resulted in arbitrary teaching of CSE and great disparities within and between schools. If the CSE program is to be successfully integrated and taught, there is a fundamental need to take local culture into account in terms of the curriculum content and teaching approaches, and to secure local ownership of the curriculum. The lack of such considerations can leave the learners at disadvantage. In Zambia, there is rapidly increasing prevalence of early pregnancy, which suggests limitations and failures in efforts aimed at addressing sexual and reproductive health challenges among adolescents. To address this problem, CSE is needed, but as this study has shown, it requires repackaging of both the content and mode of delivery with the support of teachers and other stakeholders at district level.

## Data Availability

The datasets for this study are available from the corresponding author on reasonable request.

## Abbreviations

(CISMAC): Centre for Intervention Science in Maternal and Child Health
(CSE): Comprehensive sexuality education
(ERES): Excellency in Research Ethics and Science
(IDI): In depth interview
(SAFEZT): Safe Abortion and Fertility Control in Ethiopia, Zambia and Tanzania
(SRH): Sexual and reproductive health
